# Multicolor light-emitting devices with Tb_2_O_3_ on silicon

**DOI:** 10.1038/srep42479

**Published:** 2017-02-21

**Authors:** Ling Li, Shenwei Wang, Guangyao Mu, Xue Yin, Lixin Yi

**Affiliations:** 1 Key Laboratory of Luminescence and Optical Information, Ministry of Education, Institute of Optoelectronic Technology, Beijing Jiaotong University, Beijing, 100044, China

## Abstract

Great efforts have been devoted to achieving efficient Si-based light-emitting devices. Here we report new light-emitting devices fabricated with Tb_2_O_3_ on Si substrates. Intense green electroluminescence was observed, with a turn-on voltage of about 8 V. The green emission is attributed to the characteristic transitions of Tb^3+^ ions in Tb_2_O_3_. The electroluminescence mechanisms of the Tb_2_O_3_ light-emitting devices are discussed. In addition, visible and near infrared electroluminescence was observed in rare-earth (Eu^3+^, Sm^3+^ and Yb^3+^) doped Tb_2_O_3_ light-emitting devices.

Si-based photonics have been regarded as an effective way to improve data transfer rates of information processing systems^1^. In the past decades, great efforts have been devoted to achieving efficient Si-based light sources^2^,^3^,^4^,^5^,^6^. Rare-earth (RE) doped SiO_2_ has attracted a lot of interest due to their high luminescence efficiency and wide spectral range extending from ultraviolet (UV) to infrared (IR) ranges^7^,^8^,^9^. Previously, efficient visible light-emitting devices (LEDs) based on RE-doped metal-oxide-semiconductor (MOS) structures have been demonstrated^10^,^11^,^12^. However, both the light emission yield and the reliability of the RE-doped oxides devices are limited by charge trapping and RE clustering effects^13^.

Tb_2_O_3_ is a very attractive member of RE oxides since it is a direct and wide-band-gap semiconductor (~3.8 eV), and its lattice constant (~10.73 Å) matches Si (~5.431 Å)^14^. Haugsrud *et al*. reported that Ca-doped Tb_2_O_3_ is a p-type semiconductor^15^. In particular, Tb^3+^ ions have been widely used as ideal activator ions for green display devices, detectors, and lasers^16^. In these applications, different emission wavelengths have been achieved owing to the energy transfer between Tb^3+^ and other RE ions (such as Eu^3+^, Sm^3+^, Nd^3+^, Yb^3+^)^17^,^18^,^19^,^20^. However, so far, there has been no reports on electroluminescence (EL) of Tb_2_O_3_.

Here we demonstrate, for the first time, new green LEDs using Tb_2_O_3_ on Si substrates. Tb_2_O_3_ thin films were prepared by magnetron sputtering and annealed in an O_2_ ambient. Strong green emission was observed, with a turn-on voltage of 8 V. EL mechanisms of the Tb_2_O_3_ LEDs are also discussed. In addition, intense red, orange, and near IR emissions are obtained from RE^3+^ (Eu^3+^, Sm^3+^ and Yb^3+^) doped Tb_2_O_3_ LEDs on Si substrates, respectively. Our results show that Tb_2_O_3_ LEDs can be potentially used in display, optical communication, and other Si-based optoelectronics.

## Results and Discussion

**Figure 1:**
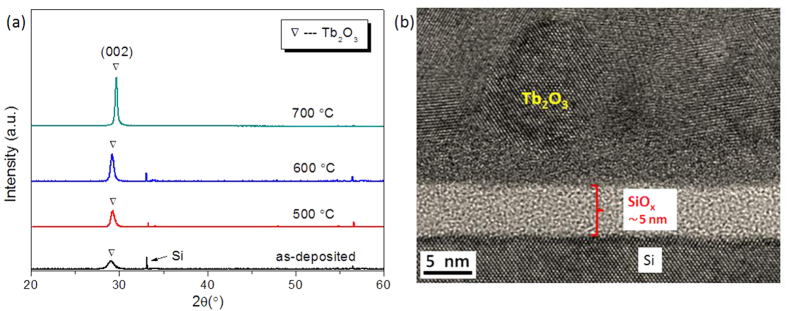
Lattice structure of the films. **(a**) XRD of as-deposited and annealed Tb_2_O_3_ films (~200 nm) at 500, 600, and 700 °C for 1 hour, respectively. (**b**) High-magnification TEM image of a Tb_2_O_3_ film.

The crystalline phases of as-deposited and annealed Tb_2_O_3_ films were investigated by X-ray diffraction (XRD). As shown in Fig. 1(a), the as-deposited film shows a weak diffraction peak at 2*θ* ≈ 29.0°, which corresponds to (002) plane of hexagonal Tb_2_O_3_. This peak increases and narrows with increasing the annealing temperature. Strong peak of (002) plane of Tb_2_O_3_ is observed when the annealing temperature is further raised to above 700 °C. The lattice structure of the films is investigated by using a transmission electron microscope (TEM). The high-magnification TEM image shows the presence of crystalline areas in the Tb_2_O_3_ film. As shown in Fig. 1(b), there is an amorphous SiO_x_ layer at the Tb_2_O_3_/Si interface, with a thickness of about 5 nm.

**Figure 2:**
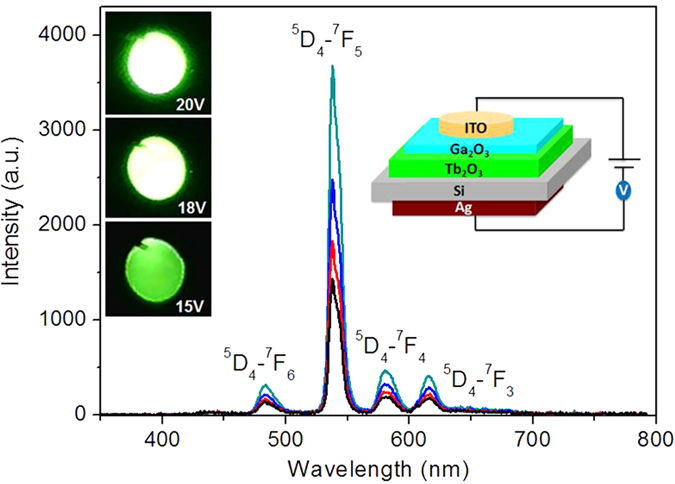
EL performance of the Tb_2_O_3_ LED. EL spectra of the LED at the voltage of 12–20 V, the insets show the structure diagram of the LED and EL photos of the device at different voltages.

A Tb_2_O_3_ LED was fabricated with the structure diagram shown in the inset of Fig. 2. Intense green EL is observed when a positive voltage is applied on the indium tin oxide (ITO) layer, while no EL is detectable under reverse biases. The turn-on voltage of the device is as low as 8 V. The emission is bright enough to be observed by naked eyes under normal room light. The EL spectrum shows peaks at 484, 540, 582, and 616 nm, which correspond to ^**5**^D_4_–^7^F_6_, ^**5**^D_4_–^7^F_5_, ^**5**^D_4_–^7^F_4_, and ^**5**^D_4_–^7^F_3_ transitions of Tb^3+^, respectively^16^. The ^5^D_4_–^7^F_5_ transition is the most intense one and features a double-peak structure, which can be attributed tothe crystal field splitting of the ground state. When increasing the forward bias, the EL spectral shape remains unchanged, with the intensity increasing with the applied voltage from 8 V up to 20 V. The forward current of the device reaches 2.8 mA when the forward bias is 20 V, while the reverse leakage current is minimal. These results show that the Tb_2_O_3_ LED has excellent rectification performance.

**Figure 3:**
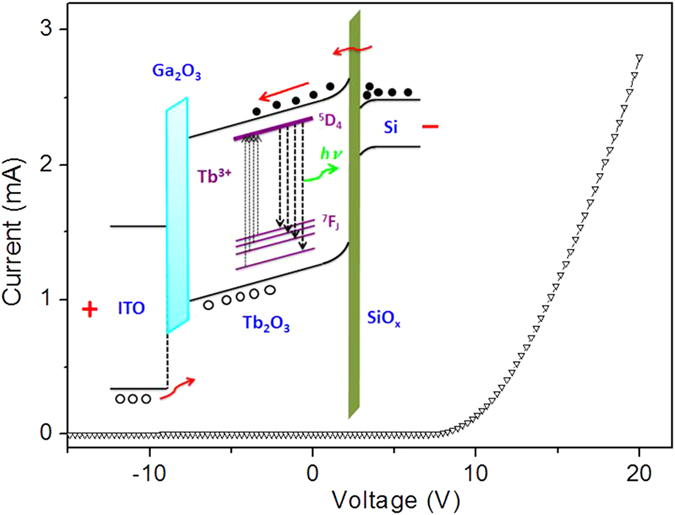
Electrical characterization of Tb_2_O_3_ LED. Current-voltage characteristic of the device. The inset shows the energy band diagram of the devices and the charge transfer process.

The EL mechanism is schematically illustrated in the inset of Fig. 3. When a sufficiently high forward bias is applied, the energy bands of both Tb_2_O_3_ and SiO_x_ bend upward along the electric field direction. According to Zhu *et al*.^21^,^22^,^23^, a trap-assisted tunneling (TAT) mechanism dominates the conduction mechanism at the EL-enabling voltages. When a sufficiently high forward bias voltage is applied between the two electrodes, a large number of electrons in Si accumulate in Si/SiO_x_ interface and then reach the conduction band of Tb_2_O_3_ by tunneling through the SiO_x_ barrier. Meanwhile, holes are injected from ITO electrode into the Ga_2_O_3_ layer and then enter the Tb_2_O_3_. The holes accumulate at Tb_2_O_3_/SiO_x_ interface due to the SiO_x_ barrier. Thus, direct impact excitation of Tb^3+^ ions are exerted when the kinetic energies exceeds the threshold energy.

**Figure 4:**
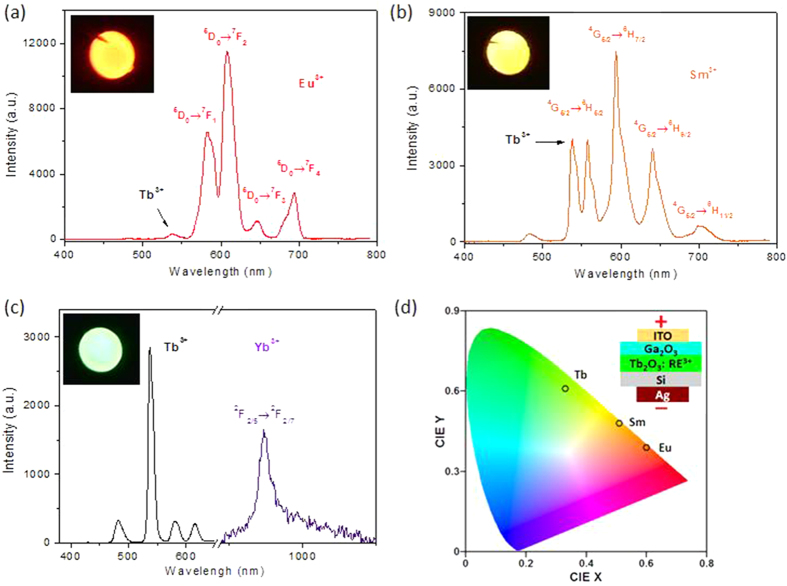
EL performance of the LEDs. (**a–c**) EL spectra of the red-, orange- and near IR-emitting devices from Eu^3+^, Sm^3+^, Yb^3+^ doped LED at forward biases of 20 V, respectively. (**d**) CIE coordinates of the green-, red- and orange-emitting devices.

We have demonstrated a bright green EL device based on Tb_2_O_3_. To further explore the possibility of using Tb_2_O_3_ as a host material to RE ions to achieve devices of other colors, we further fabricated EL devices with RE-doped Tb_2_O_3_. Intense red EL is observed from Eu^3+^ doped Tb_2_O_3_ LED. As shown in Fig. 4(a), the emission peaks are at about 582, 619, 646, and 694 nm, corresponding to ^5^D_0_–^7^F_J_ (J = 1, 2, 3, and 4) transitions of Eu^3+^. The highest peak at 619 nm corresponds to the Eu^3+^ electric dipole transitions of ^5^D_0_–^7^F_2_^17^. In Fig. 4(b), strong orange EL is observed from Sm^3+^ doped Tb_2_O_3_ LED. The characteristic of the ^4^G_5/2_–^6^H_J_ (J = 5/2, 7/2, 9/2, and 11/2) transitions of Sm^3+^ ions are appeared. The Sm^3+^ emission peaks are from transitions of ^4^G_5/2_–^6^H_5/2_ (558 nm), ^4^G_5/2_–^6^H_7/2_ (593 nm), ^4^G_5/2_–^6^H_9/2_ (640 nm), and ^4^G_5/2_–^6^H_11/2_ (701 nm)^18^. In Fig. 4(c), both green emission of Tb^3+^ and near IR emission of Yb^3+^ are obtained. The characteristic peak of Yb^3+^ is attributed to the transition from ^2^F_5/2_ to ^2^F_7/2_^19^. As shown in Fig. 4(d), The CIE coordinates of the green-, red- and orange-emitting devices are (0.33, 0.61), (0.60, 0.39) and (0.51, 0.48), respectively.

In summary, new LEDs from Si-based Tb_2_O_3_ are fabricated. Intense green EL was observed, with a turn-on voltage of about 8 V. The green emission centered at 484, 540, 582, and 616 nm, corresponding to the ^**5**^D_4_–^**7**^F_J_ transitions of Tb^3+^ in Tb_2_O_3_, where J = 6, 5, 4, and 3. The EL intensity increases with the applied voltage up to 20 V. In addition, red, orange, and near infrared EL were observed from RE^3+^ (Eu^3+^, Sm^3+^ and Yb^3+^) doped Tb_2_O_3_ LEDs, respectively. Our results could provide a possible route for achieving stable and highly efficient Si-based LEDs.

## Methods

About 200 nm Tb_2_O_3_ films and RE-doped Tb_2_O_3_ films were deposited on n-type Si (100) substrates by magnetron co-sputtering technique. The Si substrates were cleaned by dipping in a dilute HF solution (HF:H_2_O = 1:7) for 60 s. Tb (99.95%) target was sputtered in Ar:O_2_ = 15:5 atmosphere, at a substrate temperature of 150 °C. The deposition rate was 0.4 Å/s. RE ions (RE = Sm, Eu, and Yb) were doped in Tb_2_O_3_ films by sputtering with Sm (99.95%), Eu (99.95%) and Yb (99.95%) targets, respectively. Ga_2_O_3_ layer (~20 nm) was deposited by sputtering with Ga_2_O_3_ target. The as-deposited samples were annealed in O_2_ ambient at 500, 600, or 700 °C for 1 hour, respectively. We fabricated the LEDs as schematically illustrated in the inset of Fig. 2. ITO and Ag electrodes were deposited on the surface of the film and the back side of the Si substrate, respectively, both by magnetron sputtering.

The crystal structure characterization was carried out by using Bruker D8 ADVANCE XRD with Cu-Ka radiation, and the morphology of the samples was determined by TEM (Hitachi, H8100 200 kV). The EL spectra of the devices and I–V characteristics were measured by a system of an ACTON 150 CCD spectrometer and a Keithley 2410 source meter, respectively.

## Additional Information

**How to cite this article:** Li, L. *et al*. Multicolor light-emitting devices with Tb_2_O_3_ on silicon. *Sci. Rep.* **7**, 42479; doi: 10.1038/srep42479 (2017).

**Publisher's note:** Springer Nature remains neutral with regard to jurisdictional claims in published maps and institutional affiliations.
